# Cultural Adaptation and One-Year Follow-Up of the Mom-to-Mom Program Among Minority Arab Bedouin Women: Addressing Postpartum Depression

**DOI:** 10.3390/jcm14207167

**Published:** 2025-10-11

**Authors:** Samira Alfayumi-Zeadna, Anna Schmitt, Rosa Abu Agina, Ilana Schmidt, Julie Cwikel

**Affiliations:** 1 Department of Nursing Sciences, School of Health Professions, Gray Faculty of Medical and Health Sciences, Tel Aviv University, Tel Aviv 6997801, Israel; 2 Center for Women’s Health Studies and Promotion, Ben-Gurion University of the Negev, Be’er Sheva 8410501, Israel; 3 Yarrow, East Glacier Park, MT 59434, USA

**Keywords:** Arab, Bedouin, cultural adaptation, minority, Mom-to-Mom, postpartum depression

## Abstract

**Background/Objectives**: There is a growing need for programs addressing perinatal mental health, particularly for new mothers. Postpartum depression (PPD) may occur during pregnancy or within the first year postpartum, with both short- and long-term negative consequences for both mothers and their infants. This study describes the cultural adaptation, implementation, and one-year follow-up of the Mom-to-Mom (M2M) program for minority Bedouin women in Southern Israel. **Methods**: We conducted a community-based intervention (M2M) emphasizing cultural adaptation. Outreach efforts were conducted in collaboration with healthcare professionals to encourage referral to the M2M program. A total of 111 mothers completed a self-administered questionnaire that included socio-demographic characteristics and PPD symptoms (PPDs) at two time points: prior to the intervention (Time-1) and one year after participating in the program (Time-2). PPD was measured using the Edinburgh Postnatal Depression Scale (EPDS), using a score cutoff of ≥10. **Results**: There was a significant decrease in PPDs (EPDS ≥ 13) between Time-1 and Time-2 after one year of follow-up in the M2M program (from 45% to 19.8%). Of the participants, 75% were referred to the program by healthcare professionals. Among those with EPDS ≥ 10, 30% were referred to mental health services. This program provided education, professional support, and led to the establishment of the first M2M center within a Bedouin community, located in the Negev (Naqab). **Conclusions**: The results emphasize the importance of culturally sensitive approaches to increase awareness, early diagnosis, and professional support in addressing PPD, tailored to a cultural context. Culturally adapted programs can be effective in minority populations and contribute to reducing disparities in maternal mental health care.

## 1. Introduction

The transition to motherhood involves significant physical, emotional and psychological changes that may affect family dynamics [1]. Developing culturally sensitive strategies for the prevention, identification, and treatment of postpartum depression (PPD) among ethnic minority populations is critical for delivering effective care and reducing health disparities [2,3]. These strategies should consider the cultural norms, beliefs, and healthcare-seeking behaviors. Programs should incorporate the unique strengths of different cultures as protective factors in coping with PPD [4]. For instance, a study conducted in Alberta, Canada, found that cultural beliefs potential barriers to the access to and navigation of maternity services by immigrant women in Canada. The authors also demonstrated the importance of employing community health workers who share the cultural background and language of the target population [5]. These workers can help bridge the gap between healthcare providers and women from different cultures, offering culturally relevant support and guidance throughout the perinatal period. Culturally tailored interventions, including group therapy and psychoeducation delivered in the native language, have demonstrated efficacy in reducing PPD among minority populations [2]. This study showed that high PPD awareness significantly contributed to positive EPDS change in the intervention group and high social support significantly protected against negative EPDS change in both groups, intervention and control [2]. Community involvement, addressing linguistic barriers, and incorporating cultural values are essential for providing effective and equitable treatment.

PPD is a major public health concern with adverse implications for the physical and emotional health of mothers, fathers, and mother–child relationships [6]. PPD is one of the most common non-obstetric complications associated with childbearing [7], estimated to affect approximately 14% of mothers globally, with rates varying across different regions and populations [8]. Higher rates of PPD (20–45%) are reported among immigrant, indigenous, and minority women [9,10,11].

This higher PPD rates among minority, immigrant, and indigenous women linked to cultural factors, economic, and health-related factors, as well as cumulative life stress, discrimination, beyond socioeconomic status (SES) [12,13], and historical trauma and disrupted of traditional support among indigenous population [9]. Research on PPD has emphasized its high prevalence and lasting negative effects on maternal mental health, mother–child bonding, and child development [13,14]. These effects are often more severe among immigrant and minority mothers, who face limited access to social capital and resources [13]. In some populations, low awareness of PPD, language barriers, and limited access to healthcare services, and stigma barriers hinder recognition of symptoms and access to effective treatment [2]. In light of these disparities and access barriers, we focused on a culturally adapted, community-embedded peer-support model (Mom-to-Mom) and evaluated its implementation and PPD screening outcomes in the Bedouin context.

### 1.1. Background on Mom-to-Mom (M2M)

The Mom-to-Mom (M2M) program, developed by Dr. Ruth Paris in Boston, provides structured peer-support model in which trained community peers/paraprofessionals provide individual or small group support for mothers during the first postpartum year, particularly those at risk for or experiencing PPD [15,16]. Key elements of the program included emotional and practical support to new mothers, psychoeducation to promote adaptive coping, and improve access to mental health services. An evaluation in Boston showed improvements in mother–child interactions and reduced PPD [15].

Globally, para-professional home visitors from the same culture background have effectively expanded access to maternal mental health support, reduced costs, and improved outcomes [17,18]. These different modes of delivering services have been tested with nurses and using various treatment protocols, for example, using both group and individual interventions, both in person and remote [3,16,19,20]. In Israel, M2M was adapted to incorporate cultural relevance and integration with maternal and child health services. Adaptations included translated materials, community volunteers, flexible individual/group/home contacts with confidentiality/stigma-sensitive framing. It has successfully supported Jewish Israeli mothers [3,17]. However, it has yet to be tailored for the minority Arab Bedouin population in southern Israel, whose cultural and socioeconomic needs remain unaddressed.

### 1.2. Arab Bedouin Women in Southern Israel

The Bedouin community, part of Israel’s Arab minority, comprise 27.4% of the population of the Negev (Naqab), a region of Southern Israel [21]. Most (60%) live in seven government-recognized towns, while 40% live in unrecognized villages that lack essential infrastructure and access to health and social services [21]. These conditions contribute to significant health disparities [22,23]. Compared to other Israeli population groups, the Bedouin population has a lower socioeconomic status [24]. These disparities reflect broader issues such as poverty, limited healthcare access and substandard living conditions [21,24].

PPD prevalence is higher among Bedouin women (31%) compared to Arab women overall (20.8%) and Jewish women (7%) [25,26]. Barriers to treatment include poor access to services in the rural areas of the Negev (Naqab), lack of transportation, minimal screening, language barriers, low awareness, and mental health stigma [27]. Thus, this study aims to describe the cultural adaptation, implementation, and one-year follow-up of the M2M program for perinatal Bedouin women in the Negev (Naqab), focusing on the detection, prevention, and treatment of PPD, and the role of culturally sensitive interventions in improving maternal mental health outcomes. We hypothesized that M2M would be feasible to deliver and that most mothers would screen below EPDS ≥ 10 at one year, documenting these outcomes in a minority community contributes practice-based evidence with direct clinical implications for integrating culturally responsive peer support into routine maternal–child health services to improve detection, referral, and support for mothers at risk for PPD in underserved settings.

## 2. Materials and Methods

### 2.1. Sample and Data Collection

The sample comprised 111 mothers between 18 and 45 years of age, who were referred to the M2M program via health professionals (nurses, physicians, social workers) or self-referral (friends, family, internet). Mothers were referred if they were facing challenges in coping with motherhood or were experiencing PPD symptoms (PPDs). All women joined M2M between February 2021 and September 2023 and completed depression screening between 1 and 12 months postpartum (35% completed the screening in 1–3 months, 34% completed the screening in 4–6 months, 20% completed the screening in 7–9 months, and 11% completed the screening 10–12 months postpartum), and one year after joining the program. With n = 111 participants, we have 89% power to detect a reduction in EPDS ≥ 10 prevalence from 31% to 18%; therefore, we focus on effect sizes and 95% confidence intervals. A total of 111 mothers enrolled in M2M and completed the baseline questionnaire. At one-year follow-up (Time-2), 99/111 (89.2%) completed the EPDS assessment, and 12/111 (10.8%) were lost to follow-up.

### 2.2. Data Collection

#### Time 1 Interview

At this stage, 111 women were recruited, and after participants granted informed consent, they were administered an online, structured, self-reported questionnaire in Arabic. All mothers joined M2M after an initial intake meeting with the project leader (via phone or face-to-face) and were directed to the project’s website, where they could complete the online questionnaire. All participants received an explanation in Arabic about M2M and direct contact information, such as telephone numbers, for clinic staff and support services. The intervention began when the women were one month to one year postpartum.

### 2.3. Study Measures

The following measures were all included in the online self-reported questionnaire.

*Sociodemographic Characteristics*: We asked women their age (15–24, 25–34, and ≥35), type of residence (recognized/unrecognized), number of children (0–1, 2–3, and ≥4), education (non-academic/academic degree), and employment status (currently employed/unemployed), and polygamous marriage (no/yes) (was defined as a marital arrangement in which a man has more than one wife, as reported by the participant).

*Health Variables*: Health variables included pregnancy complications (yes/no), childbirth complications (yes/no), number of months postpartum, current chronic disease (yes/no), and history of PPD (yes/no) was defined as self-reported prior clinician-diagnosed PPD and/or treatment (psychotherapy and/or antidepressant medication) for postpartum. All mothers were asked about their medical history, even if they were joining with their first child, to account for previous pregnancy or miscarriage. Gestational diabetes, gestational hypertension, preeclampsia, multiple pregnancy, anemia, placenta previa, or bleeding during pregnancy were considered pregnancy complications. Childbirth complications were defined as Cesarean section complications, postpartum hemorrhage, fetal distress, or infection, and Baby’s Age (months).

*Postpartum Depression*: PPD was measured using the 10-item EPDS screening which asks women to report the frequency of various depressive symptoms over the past week. The validated Arabic version of the EPDS was utilized. The Arabic version of the EPDS has been widely used among Bedouin women in Israel [26]. The EPDS score (continuous EPDS), which ranged from 0 to 30, was dichotomized using the ≥10 cutoff, where EPDS ≥ 10 indicated presence of depressive symptoms [28]. As with any screening instrument, EPDS scores denote the possible presence of the disorder, and do not constitute a clinical diagnosis. All of the mothers who scored 14 and over and/or were positive on question 10 (regarding self-harm) were referred to a mental health professional. It should be noted that any positive response on question 10 (self-harm) requires immediate attention by a mental health professional [29]. In this study, internal consistency (Cronbach’s α) of EPDS responses during pregnancy and postpartum was 0.865.

### 2.4. M2M Program (Intervention)

Cultural Adaptation of the M2M Program: Various cultural adaptations of M2M program were made before the program was initiated among the Bedouin community of southern Israel. These adaptations included building a culturally relevant staff, materials adaptations and translations, trust-building, community connectivity, and role reconfiguration, as detailed below. Linguistic adaptations were made for program materials, such as brochures and intake questionnaires, which were translated into Arabic. The professional and paraprofessional staff actively built relationships with Bedouin community leaders and professionals, including gynecologists, psychiatrists, psychologists, social workers, nurses, and religious leaders. These connections fostered trust and cooperation, enabling bi-directional referrals and program support. The M2M team consisted of both professional and paraprofessional staff, including social workers, nurses, and community-based outreach workers. In addition to the questionnaires, we conducted weekly staff meetings, where team members reviewed outreach and treatment issues.

M2M Program Implementation and Community Integration: The M2M program established numerous activities and partnerships to assist perinatal Bedouin women during its cultural adaptation and implementation process. Table 1 shows the key activities and collaborations of the M2M program from 2021 to 2023, including group meetings, community events, and partnerships that were designed to provide support, education, and culturally adapted services to Bedouin women.

Formation of the M2M Center for Bedouin Women: Through this project, a M2M Center for Bedouin women established in a central Arab Bedouin village in southern Israel for the first time with a strategic focus on the needs of perinatal Bedouin women. The center is co-located within an Early Childhood Center and Maternal Child Health (MCH) clinic. These cultural adaptations ensured that the program was culturally relevant, acceptable to the local population and effective. Real-life experiences, education, and research of staff members were critical factors in achieving cultural adaptation, as they provided the necessary understanding and tools to navigate the complexities of this diverse cultural context, starting with how to enroll mothers in the program.

M2M Group Meeting: Eight groups were conducted with Bedouin mothers over the course of 2 years. There were 3–5 sessions per group and 5–9 women participated in each group. These meetings were held in various formats and frequencies to offer education, support, and/or recommendations about treatment for PPD. Other topics covered were positive parenting practices, postpartum medical follow-up, encouraging healthy nutrition and nutritional supplements (e.g., iron, B12, vitamin D), breastfeeding, exercise, and positive mental health practices. During the COVID-19 period (2021) 3 groups were conducted via Zoom, with 5–6 women participating in each group. There were five groups that met in person, all of which comprised 6–9 women. The in-person meetings occurred in various Bedouin towns and communities. One group met in the Maternal and Child Health Center in location S, while other groups were gathered at community centers in the location L and location R.

Community Events: Four events were held for the broader community and were not limited to program participants. This helped to raise awareness and knowledge about the postpartum period (PPD specifically) and to integrate the community into the support process. The first event was presenting the International Mothers Mental Health Day Conference, which aimed at raising awareness about perinatal mental health issues worldwide. The second event was the first-ever women’s health meeting to include male religious leaders, which engaged different community leaders in addressing cultural and religious aspects of women’s mental health issues. The third event consisted of participating in the Bedouin Women’s Health Conference (organized by the local council), which served as a platform to discuss various aspects of women’s mental and physical health and involved multiple stakeholders from the community and other health sectors. The fourth event was held with a group of women participating in a program for community leaders. Topics covered at this event included women’s mental health, postpartum depression, and genetic disease in the Bedouin community. All four community events were made possible through collaboration with community partners.

Community Partnerships: Partnerships with a variety of local community and health organizations were formed. Partnerships enabled comprehensive care for participants through the integration of services and the establishment of a network of care. Partnerships were established or expanded with the local hospital, regional gynecologists, psychiatrists, community clinics, community centers, and one maternal and child health center (MCHC). Partnerships enabled referral, shared care, and enabled a more holistic care in addressing women’s health concerns, including mental health. Additionally, the M2M team recorded several podcasts on PPD. One was recorded in Arabic and was conducted by the project leader and one of the paraprofessional members of the team, both of whom are from the Bedouin community. This podcast was for the community and focused on PPD symptoms, risk factors, and treatment options. Another podcast was recorded in Hebrew for professionals (social and health workers) that outlined unique characteristics of PPD among Bedouin mothers and the cultural adaptations used in M2M, Such as Arabic-language materials, female Bedouin professionals/paraprofessionals, choice of individual or group support (home/clinic/telephone) with confidentiality and stigma-sensitive framing, and formal referral/safety pathways in collaboration with MCHCs/Early Childhood Centers/the regional hospital. This podcast was conducted by the project leader and a social worker from the team. These podcasts were used to train social work students and were made available to the community of mental health and reproductive health professionals. In summary, these activities served to increase knowledge, support, and consultation while providing a bridge between services to ensure a broad-based and culturally sensitive intervention (Table 1).

### 2.5. Program Delivery

Staff Roles and Leadership: The professional staff included the project leader (first author), who is a nurse and PhD public health professional with extensive experience and in women’s health within the Bedouin community. The paraprofessional staff, also Bedouin, had background in women’s health as well as experience working with Bedouin women. They led the M2M groups across various locations.

Program Execution: Every group session was conducted in Arabic by a professional or paraprofessional Bedouin woman. Paraprofessional or professional contacts with participants were made via phone or by home visits, with home visits offered only when deemed appropriate by the new mother. Otherwise, meetings were held at the local mother–child health center.

Program Content and Support: The program engaged Bedouin women in a culturally appropriate way across various locations, offering knowledge, personal and group support, and/or referral to professional consultation and treatment. A comprehensive educational approach covered key aspects of maternal mental and postpartum health including PPD, positive parenting practices, medical follow-ups, nutrition and nutritional supplements (e.g., iron, B12, vitamin D), breastfeeding, exercise, mental health practices, and contraception.

Modes of Participation: Women could choose between one-on-one or group sessions and had the option to engage in the program through Zoom, phone, or in person meetings.

Time-2 Interview: Women who participated in the study at Time-1 were invited to complete a second online EPDS assessment one year after starting the intervention (M2M program).

Statistical Methods: Quantitative data from the questionnaires were analyzed using SPSS software version 28. To explore the associations between sociodemographic and health characteristics and PPDs, we conducted standard bivariate analyses using *t*-test and chi-square tests. Independent variables significantly (*p* < 0.05) associated with EPDS ≥ 10 in the univariate analysis were included in the multivariable logistic regression analysis to examine predictors for PPDs.

Ethical Considerations: This study was approved by the Ethics Committee of the Be Gurion University of the Negev (approval number 3/2021/14-A, date February 2021).

## 3. Results

Table 2 shows the demographic and health characteristics of the study participants. The average age of participants was 29.5 (Standard Deviation (SD = 6.3), with 87.4% of participants residing in a recognized village. On average, participants had 3 children (SD = 2), 25.2% with their first child and 22.5% with more than four children. On average, participants had completed high school (13.2 years of education (SD = 3.1), while 29.7% pursued higher education. Only 35.8% were employed. 14.4% were in a polygamous marriage. A small proportion (6.3%) of participants reported becoming pregnant through IVF, 7.3% reported having a chronic disease or condition, 23.4% experienced pregnancy complications, and 24.3% had childbirth complications. At the time of the interview, 49.5% of the mothers were breastfeeding, either exclusively or partially. 11.7% of mothers reported a history of PPD.

The mean EPDS score among participants at Time-1 was 9.7 (SD = 5.9). According to the EPDS scores, 45% of participating mothers showed symptoms of PPD (10 and over). Nine mothers (8.1%) reported a positive result on the self-harm question. EPDS score distributions were compared using sociodemographic parameters based on socioeconomic and health characteristics. Older women, women in polygamous marriage, mothers who reported pregnancy complications, childbirth complications, or a history of PPD had significantly higher rates of PPDs with this current birth than did their counterparts. Other demographic variables were not found to have a statistically significant relationship with the EPDS score (Table 3).

Table 4 presents the results of a multivariate logistic regression model comparing women who had EPDS scores ≥ 10 to women who scored lower than 10 (Time-1). Childbirth complications were associated with PPDs. Women who experienced childbirth complications were 3.4 times more likely to develop depressive symptoms. There were no differences in the distributions of EPDS scores between age, polygamy, and history of PPD.

Table 5 shows the frequency of subsequent referrals from M2M to a higher level of professional care. Over a quarter of those in the program were referred to additional consultations with medical or psychiatric professionals and over 80% of those referred received care, all of these women had EPDS scores in the clinical symptom range. The follow-up conducted one year after participation in the M2M program (Time-2) revealed that 69% (*n* = 77) of mothers had an EPDS < 10. These mothers reported they were well and functioning at home and at work. 19.8% of mothers reported PPDs (EPDS ≥ 10) at Time-2. At Time-2, no participants reported a positive response to question 10 regarding self-harm. 12 mothers were lost to follow-up.

## 4. Discussion

This study aimed to describe the cultural adaptation, implementation, and one-year follow-up of the M2M program for Bedouin women in southern Israel. This is the first study to evaluate the M2M program within the indigenous Arab population, and in particular within the Bedouin community of southern Israel. The study sheds light on the prevalence and severity of PPDs among participants referred to M2M by professionals (75%) or self-referral (25%). 45% of these mothers showed symptoms of PPD. Mothers were referred if they were facing challenges in coping with motherhood or were experiencing PPDs. The M2M program effectively addressed these challenges through culturally tailored support, education, and referrals. This is evidenced by lower EPDS scores one year after program participation (19.8% vs. 45%). These findings confirm previous research findings from the M2M program among Jewish mothers in southern Israel, which indicated that 73.6% of mothers were referred by a medical or social professional, with 38.7% having scores of EPDS ≥ 10 [3].

In our study, 45% of participating mothers showed PPDs and 29% of participants had results suggesting moderate to severe PPDs. Other studies have reported higher rates of PPD (20–49%) among women from ethnic minority populations [9,10,11]. In addition, nine mothers (8.1%) reported thoughts of self-harm on the EPDS questionnaire. Our baseline estimate reflects mothers who self-referred or were referred to a support program due to distress, so the incidence of entry is likely to be higher than in population-based samples. Previous studies in Israel have reported lower rates, ranging from 1–3% in an early study in a low socioeconomic area [26,30]. Globally, postpartum suicidal ideation has been reported in 2–11% of women [31,32]. Suicidal ideation and attempts are among the most severe consequences of PPD, compounding the emotional and psychological impact on mother, infant, and the families [33]. At Time-2 assessment (12 months postpartum) the observed rate was lower, a timing likely contributing to the lower rate observed. In addition, the group included mothers who participated in a supportive program, which may have reduced distress among those who remained in the study. In the current study, mothers who reported pregnancy and childbirth complications and a history of PPD had significantly higher rates of PPDs. Women who experienced childbirth complications were 3.4 times more likely to develop depressive symptoms. Studies conducted among Bedouin and Jewish women in southern Israel indicated that 7% to 10.3% experienced pregnancy complications [34,35]. A longitudinal study showed that childbirth complications were associated with chronic PPD [36]. Similar results have been reported in additional recent studies among Bedouin women [2] and other Jewish Israeli mothers [36].

The cultural adaptation process was a critical aspect of the M2M program’s success in addressing PPD among Bedouin women. Because this study did not include qualitative instruments, we refrain from drawing inferences about mechanisms (e.g., trust building, stigma reduction) underlying engagement. Any references to these contextual elements are presented only as hypotheses grounded in prior literature and our program description (Table 1) and are not reported as outcomes of the current study. In this service-embedded program, cultural adaptations were implemented to align delivery with Bedouin social norms and language. In Bedouin society, cultural norms emphasize privacy, honor, and protecting the family’s reputation, leading to reluctance in sharing personal problems with others, especially those outside designated health or professional roles bound by confidentiality [37,38]. Another key cultural aspect must be highlighted, all of which center around trust. As with many communal, indigenous societies [39], establishing and maintaining trust is central to relationship building among the Bedouin. The preservation of culturally acceptable routes to trust building also helped women to open up about mental health, which is not commonly discussed in the Bedouin community [27].

Mothers could choose individual support, group support, or both, and services were offered in Arabic with translated materials. This cultural context affects how support services are provided and received, making cultural sensitivity a crucial aspect of the M2M program’s approach [40]. Additionally, ensuring therapies were culturally relevant and included translations was critical for making a M2M program acceptable to participants and integrating it into the health system. Various studies have also highlighted the importance of culturally adapting perinatal interventions to increase their effectiveness [41,42].

Partnerships with MCHCs, Early Childhood Centers, and the regional hospital, leading to strong connections within the Bedouin and the medical community. Studies found that cultural adaptations through collaboration with community members and maternal health providers, improved health outcomes and reduced disparities [43,44]. Community engagement included educational sessions on PPD with religious Bedouin leaders, aiming to increase awareness and reduce stigma, marking the first-time PPD was discussed with this group. This aligns with studies showing that positive relationships and culturally appropriate interventions help increase awareness and reduce stigma around PPD [2,45]. Building relationships across the community and among healthcare professionals, allows a more comprehensive approach to addressing PPD.

Group meetings facilitated the development of social networks and provide opportunities for women to gather in a way that might not otherwise have been possible, particularly through meetings at the newly established M2M center in the Bedouin community [3,16,17]. Although such features are theoretically consistent with effective peer support, our data do not permit causal inference about their specific impact.

This project represents a pioneering effort to adapt and implement a culturally tailored M2M perinatal support program for Bedouin women in Southern Israel. By addressing the unique cultural, socioeconomic, and societal realities of the Bedouin community, this study not only highlights the prevalence and severity of PPD among participating mothers but has also provides valuable insights into the importance of culturally sensitive interventions. The program’s cultural adaptations, including the involvement of trusted community members, flexible support options, and initiatives to raise awareness about maternal mental health, helped build trust and reduce the stigma surrounding PPD within the community. Furthermore, this project laid the groundwork for collaboration with local organizations and led to establishment of a dedicated M2M center, marking a significant step toward comprehensive maternal mental health support to Bedouin women and their families. Overall, this study emphasizes the importance of culturally relevant interventions in promoting maternal mental health and well-being, particularly in the perinatal period. By integrating cultural values and traditions into healthcare systems, such interventions can help address psychosocial challenges, reduce disparities, and contribute to healthier outcomes for mothers, infants, and their families in diverse communities.

### Limitations

This study had some limitations. First, this study did not include a control group. Although a control group would have strengthened the ability to assess the program’s impact, in this cultural context a non-treatment control group was not ethically or professionally feasible. Second, because participation and one-year follow-up were voluntary, selection bias is possible, the findings reflect mothers who engaged with the program and should not be over-generalized or interpreted causally. Third, the using self-reported questionnaires to assess PPDs may not always accurately reflect the participants’ experiences or mental health status. Longitudinal studies that follow participants over time would provide a better understanding of the trajectory of PPD and the long-term effects of the M2M program. Fourth, the EPDS is a screening, not diagnostic instrument, EPDS-based estimates may include false positives, thus, the reported prevalence should be interpreted with caution. Fifth, because group participation was optional and some mothers received only personal or mixed contacts, the exposure method and dose varied, we did not stratify the results by exposure, which limited inference specific to the exposure method. Finally, we did not collect qualitative data (e.g., participant interviews, focus groups) or validated measures of constructs such as trust, cultural safety, or stigma; therefore, we cannot confirm these mechanisms within the present dataset, and any related statements should be interpreted as contextual hypotheses rather than empirical findings. Future studies should incorporate mixed methods designs combining quantitative outcomes with qualitative approaches and validated implementation/experience measures to rigorously test whether culturally tailored components (e.g., culturally concordant staffing, language adaptations, community partnerships) drive engagement and reductions in PPD symptoms.

## 5. Conclusions

This study emphasizes the high prevalence of PPD among Arab Bedouin women in the Negev (Naqab), with 45% of participants reporting PPDs. Through culturally tailored support, education, and referrals, the M2M program effectively addressed these challenges, leading to a reduction in PPDs from 45% to 19.8% at one-year follow-up. Increasing awareness, providing professional support, and promoting early identification are essential components in the prevention of PPD. The M2M model has the potential to expand access to care and reduce stigma surrounding PPD. Offering multiple models of engagement telephone, Zoom, and in-person individual or group sessions proved particularly important in meeting the diverse needs of participants. Implications for practice include the development and implementation of culturally tailored support programs, promotion of early screening and awareness, stigma reduction, and fostering collaboration between healthcare professionals and the community to ensure comprehensive support for mothers experiencing PPD. Future interventions should focus on further enhancing PPD prevention and support services for mothers, fathers, and infants. Future research directions should include a control group, and effectiveness should be tested in comparative designs (e.g., cluster randomization, or matched controls, test dose–response and modality-specific effects) with a longer follow-up. In addition, qualitative research, such as in-depth interviews or focus groups, may offer deeper insights into mothers’ experiences, satisfaction, and the impact of the M2M program.

## Figures and Tables

**Table 1:** Summary of M2M Activities: Group Meetings, Community Events, and Partnerships (2021–2023).

Category	Description	Details
M2M Center	Establishment of M2M Center for Bedouin Women	For the first time, a M2M Center was established in a Bedouin village. Located within an Early Childhood Center and a Maternal Child Health clinic.
M2M Group Meetings	Total groups conducted over two years (2021–2023)	8 groups, 3–5 sessions per group, 5–9 women per group. Topics: PPD, parenting, nutrition, breastfeeding
	Groups conducted via Zoom during COVID-19 (2021)	3 groups, 5–6 women per group Topics: education, support, PPD treatment, positive parenting practices, postpartum medical follow-up, encouraging healthy nutrition and nutritional supplements, breastfeeding, and exercise.
	Meetings conducted in-person in different Bedouin villages (2021–2023)	5 groups, 6–9 women per group. Locations: S, L, R Topics: support, PPD treatment, positive parenting practices, postpartum medical follow-up, encouraging healthy nutrition and nutritional supplements, and breastfeeding.
Community Events	International Mothers Mental Health Day Conference	Focused on perinatal mental health awareness
	First-ever meeting with men religious leaders on women’s mental health	Engaged religious leaders to address cultural aspects of mental health
	Participation in Bedouin Women’s Health Conference	Platform to discuss women’s health issues with community stakeholders
	Event for women participating in a program for community leaders	Focused on mental health, PPD, and genetic diseases in the Bedouin community
Community Partnerships	Partnerships with local health and community institutions	Included hospitals, gynecologists, psychiatrists, clinics, community centers
	Podcasts on PPD for Bedouin community and professionals	Recorded in Arabic and Hebrew, focused on PPD, cultural adaptation.

**Table 2:** Sociodemographic and health characteristics of the participants in the Bedouin M2M program.

Characteristics	N = 111 (100%)
Mother’s Age (Years) (Mean (SD), Range)	29.5 (6.3), 18–45
18–25	37 (33.3)
25–35	50 (45.0)
≥36	23 (20.7)
Missing	1 (0.9)
Residence Type	
Recognized village	97 (87.4)
Unrecognized village	14 (12.6)
Number of Children (Mean (SD), Range)	3 (2), 0–11
1 (First Child)	28 (25.2)
2–4	58 (52.3)
>4	25 (22.5)
Education (Mean (SD), Range)	13.2 (3.1), 5–24
<12 years of education	78 (70.3)
Higher education	33 (29.7)
Employment	
Employed	39 (35.1)
Unemployed	72 (64.9)
Polygamy	
No	94 (85.5)
Yes	16 (14.4)
Pregnancy Type	
Spontaneous	104 (93.7)
IVF	7 (6.3)
Pregnancy Complications *	26 (23.4)
Childbirth Complications ^	27 (24.3)
Baby’s Age (months) (Mean (SD), Range)	4.8 (3.4), 1–12
1–6	77 (69.4)
7–12	34 (30.6)
Breastfeeding (Full/partial at the time of the interview)	55 (49.5)
Chronic Disease (any chronic condition)	8 (7.2)
History of Postpartum Depression	13 (11.7)

* Gestational diabetes, gestational hypertension, preeclampsia, multiple pregnancy, anemia, placenta previa, or bleeding during pregnancy. ^ Cesarean section complications, postpartum hemorrhage, fetal distress, or infection.

**Table 3:** Selected sociodemographic and health characteristics of the study participants, stratified by EPDS scores (Time 1) (N = 111).

Characteristics	EPDS < 10 61 (55.0%)	EPDS ≥ 10 50 (45.0%)	*p*-Value
	N (%)/Mean (SD)	N (%)/Mean (SD)	
Mother’s Age (Years)	28.7 (5.3)	30.7 (7.3)	0.050
Residence Type			0.453
Recognized village	52 (53.6)	45 (46.4)	
Unrecognized village	9 (64.3)	5 (35.7)	
Number of Children	3 (1)	3 (2)	0.458
Education			0.436
High school	41 (52.6)	37 (47.4)	
Higher education	20 (60.6)	13 (39.4)	
Employment			0.863
Employed	21 (53.8)	18 (46.2)	
Unemployed	40 (55.6)	32 (44.4)	
Polygamy			0.039
No	56 (58.9)	39 (41.1)	
Yes	5 (31.3)	11 (68.8)	
Pregnancy Type			0.365
Spontaneous	56 (53.8)	48 (46.2)	
IVF	5 (71.4)	2 (28.6)	
Pregnancy Complications *			0.303
No	49 (57.6)	36 (42.2)	
Yes	12 (46.2)	14 (53.8)	
Childbirth Complications ^			0.002
No	53 (63.1)	31 (36.9)	
Yes	8 (29.6)	19 (70.4)	
Baby’s Age (months)			0.267
1–6	45 (58.4)	32 (41.6)	
7–12	16 (47.1)	18 (52.9)	
Breastfeeding (Full/partial at the time of the interview)			0.150
Yes	34 (61.8)	21 (38.2)	
No	27 (48.2)	29 (51.8)	
Chronic Disease (any chronic condition)			0.077
No	59 (57.3)	44 (42.7)	
Yes	2 (25.0)	6 (75.0)	
History of Postpartum Depression			0.014
No	58 (59.2)	40 (40.8)	
Yes	3 (23.1)	10 (76.9)	

* Gestational diabetes, gestational hypertension, preeclampsia, multiple pregnancy, anemia, placenta previa, or bleeding during pregnancy. ^ Cesarean section complications, postpartum hemorrhage, fetal distress, or infection.

**Table 4:** Factors associated with PPDS (Time-1), results from a multivariate logistic regression analysis (N = 111).

Variable	OR	CI 95%	*p*-Value
Age	1.047	0.982–1.117	0.163
Polygamy			0.118
No	1		
Yes	2.627	0.783–8.817	
Childbirth Complications			0.018
No	1		
Yes	3.407	1.235–9.398	
History of Postpartum Depression			0.229
No	1		
Yes	2.475	0.565–10.840	

**Table 5:** Referral Sources and PPDs Follow-Up (Time-2) in M2M Program (N = 111).

Variables	N (%)/Mean(SD), Range
Referral to M2M	
Self-referral/friends/family/internet	31 (24.9%)
Referral from professionals	80 (75.1)
Referrals to higher levels of care	
Referrals from M2M to professional consultation (psychiatric)	32 (28.8%) with clinical PPDs
Mothers who received professional consultation and treatment (care)	26 (23.4%)
PPDs (Time 2) 1 year after participation in the M2M program (N = 99)	Mean = 7.2 (5.1), Range = 0–27
EPDS < 10	77 (69.4%)
EPDS ≥ 10	22 (19.8%)
Lost to follow-up	12 (10.8%)

## Data Availability

The data supporting the findings of this study are available from the corresponding author upon reasonable request.
